# Pain Asymbolia as Depersonalization for Pain Experience. An Interoceptive Active Inference Account

**DOI:** 10.3389/fpsyg.2020.523710

**Published:** 2020-10-29

**Authors:** Philip Gerrans

**Affiliations:** Department of Philosophy, University of Adelaide, Adelaide, SA, Australia

**Keywords:** interoception, self-awareness, depersonalization, predictive processing, pain asymbolia

## Abstract

“Mineness,” also called “subjective presence” or “personalization,” is the feeling that experiences belong to a continuing self. This article argues that mineness is produced by processes of interoceptive active inference that model the self as the underlying cause of continuity and coherence in affective experience. A key component of this hierarchical processing system and hub of affective self-modeling is activity in the anterior insula cortex. I defend the account by applying it to the phenomenon of pain asymbolia, a condition in which nociceptive signals (of bodily damage) are not attributed to the self. Thus, pain asymbolia is a form of “depersonalization for pain” as Klein puts it. The pain is experienced as happening to *my body* but is not experienced as *mine*. Thus, we can describe it as loss of subjective presence or “mineness” for the experience of pain.

## Introduction

The topic of this article is a form of experience variously baptized “subjective presence,” “mineness,” or “personalization” (Seth et al., 2011; Seth, 2013; Billon, 2017a; Guillot, 2017). As the name suggests, it refers to the feeling that experiences belong to a continuing self or comprise autobiographical episodes. The nature and even existence of this elusive phenomenon are contested. However, one important reason for thinking that this form of experience is a genuine phenomenon is a pathological condition in which subjects claim that experiences feel as though they do not “belong to them.” In such cases, subjects are not in doubt that they are the subject of experience, sensory, bodily, or cognitive, but they report feeling as though the experience is not “theirs.” Such experiences comprise the essence of disorders of depersonalization. These disorders suggest that the feeling of mineness has a distinct phenomenological signature, which can be lost in some conditions, and invites investigation of its causes and typical and atypical manifestation.

As Alexandre Billon puts it:

“Every sensation has such a coefficient; we do not notice it, always encountering it; we need to be confronted with exceptional and pathological cases (…) to notice it and measure its importance (Billon, 2017a).

As a way to develop an account of this experience, I focus on a condition called pain asymbolia, aptly described by Klein (2015) as “depersonalization for pain.” In pain asymbolia, subjects report feeling detached from painful experience as though it is happening in their body but is not “theirs.” It presents as a case of loss of “mineness” for the experience of pain. Pain asymbolia is of particular interest because pain is a bodily state that is normally felt as urgently belonging to the self. For example, Descartes thought it as one of the bodily states that “teach me that… I compose a single thing with it [my body].” Wittgenstein’s anti-Cartesian meditations on pain were devoted to explaining the intuition that one could not be in doubt that one was the subject of painful experience. “It is nonsense to say that “I know I am in pain” as it means nothing more than that “I am in pain” (Philosophical Investigations 246).

The account of pain asymbolia I provide situates it in the framework of active inference theories of embodied selfhood, emotion, affect, and self-awareness (Friston et al., 2011; Limanowski and Blankenburg, 2013; Pezzulo et al., 2015; Barrett et al., 2016; Seth and Friston, 2016; Kirchhoff et al., 2018). One version of that framework is proposed by Hohwy and Michael (2017). They argue that experience of embodied selfhood is the product of an inference about the hidden causes of interoceptive (representation of states of the internal *milieu*) experience. On their view, the mind integrates signals from disparate interoceptive channels by inferring that they have a common origin in a unified entity: a bodily self.

This account forms part of an active inference account that treats cognition and action as a hierarchically integrated suite of processes whose goal is reduction of variational free energy. On this account, cognition is the iterative use of generative models (representations) to predict the consequences of actions taken to optimize organismic functioning (Friston, 2010; Hohwy, 2013; Pezzulo et al., 2015). Discrepancies between predicted and actual sensory consequences of action, signaled as prediction errors, entrain the next round of action to reduce error or optimize the model. The active inference theory tells us that prediction error is best minimized over the long term by attributing internally generated interoceptive sensations to a stable, unified entity, a self (Limanowski and Blankenburg, 2013; Hohwy and Michael, 2017; Letheby and Gerrans, 2017). This process of self-modeling creates a basic bodily form of self-awareness.

As Anil Seth puts it:

“Mental representations of selfhood are ultimately grounded in representations of the body, with the internal physiological milieu providing a primary reference—a ‘**material me**”’ (Seth, 2014).

As suggested by Seth, *material me* provides an anchor for other forms of self-representation. Sensorimotor control, agency, perceptual perspective, and explicit narrative self-representation are cognitive processes that require a form of implicit or explicit self-representation: a model of the entity that sustains the relevant process. The basic sense of being the continuing subject of experience on whose behalf all these activities are performed is the awareness of material me and underpins other forms of self-representation and awareness.

The explanation of mineness and its absence in depersonalization I propose focuses attention on a particular form of interoceptive self-representation, namely, *affective* self-representation. In affective self-representation, the mind models the bodily self as the source of affective experience and target of affective regulation. This level of self-modeling produces the experience of being the person/entity whose affective states modulate as her goals are realized or frustrated in action. We can call this the experience of being ***affective me***.

At still higher levels of self-modeling, we deploy explicit conceptual or imagistic representations of the self. This is the level at which we self-attribute character and personality traits using a self-concept. We can call this self-model **narrative me** because it models the self as the protagonist of a recountable autobiography (Schechtman, 2011; Goldie, 2011).

Affective me and interoceptive me are very closely related, because affective experience is a form of interoceptive experience. However, they are distinguishable, particularly in conditions such as depersonalization in which they dissociate. These conditions do not seem to be the result of loss of interoceptive or other basic capacities for body sensation and regulation. Rather, I shall argue that depersonalization is the result of a failure of affective self-modeling resulting from impairment in the neural substrates of affective me. Someone with depersonalization experience has the intractable experience of an intact material and narrative me, combined with hypoactivity in the circuitry that sustains affective me.

The concept of an affective me produced by interoceptive active inference integrates and synthesizes ideas advanced in different forms by a variety of theorists across disciplines of psychiatry neuroscience cognitive science and philosophy. I first explain the concept of interoceptive active inference and show how it explains (i) how interoception and affective experience are related via the process of hierarchical self-modeling, (ii) how the framework explains the role of the anterior insula cortex (AIC) in producing the experience of “mineness,” “subjective presence,” or “personalization” of experience. I then apply the framework to the explanation of pain aysmbolia. Pain asymbolia is a case in which nociceptive signals (of bodily damage) are not integrated with affective signals because of hypoactivity in the anterior insula. The mind, however, *predicts* that affective me will feel distress as a consequence of pain/nociception. The result is a prediction error that cannot be resolved because relevant affective and self-modeling mechanisms are deactivated. As a result, when an experience of pain, predicted to have a strong affective signature, does not produce affect, the subject feels as though it is not happening to her.

## Material Me: Interoception as Allostatic Active Inference

Interoceptive and affective states have a common basis in allostatic (action to optimize internal body states in context) regulation. Allostasis is a refinement of the concept of homeostasis, which implies a reflexive return to an optimal “set point” for levels of basic bodily function such as blood oxygenation. Allostasis extends that concept, recognizing that optimality for some variables requires variation according to context rather than maintenance of a single optimal set point. In fact, it can be helpful to think of homeostasis and allostasis as on a continuum of flexibility. Some functions (like blood oxygenation) have very tight parameters and are context insensitive. Others (like blood pressure) need to fluctuate more widely to sustain viability of the organism. Thus, some variables representing body state are monitored not only in relation to a homeostatic set point, but also in relation to their departure from a level predicted as optimal in context (Barrett and Simmons, 2015; Barrett et al., 2016; Corcoran and Hohwy, 2017; Kleckner et al., 2017). Allostasis thus introduces an element of forecasting to homeostatic regulation.

“Interoception and homeostatic regulation are inevitably linked and form a closed loop: tuning the set points of homeostatic reflex arcs depends on accurate allostatic predictions about future bodily states; these predictions, in turn, depend on accurate inference about current bodily states” (Stephan et al., 2016).

Interoception is the integrated representation of information about states of very basic, dynamically controlled, bodily processes such as blood oxygenation and endocrine and electrolyte balance for the purpose of allostatic regulation. In order to regulate the body, interoception models the hidden causes of allostatic fluctuations by attributing them to a unified entity (Limanowski and Blankenburg, 2013; Moutoussis et al., 2014; Sel, 2014; Barrett, 2017; Seth and Tsakiris, 2018; Wiese, 2018). The unified hidden cause of allostatic variation along multiple dimensions tracked and integrated in interoception is material me.

Interoception effectively integrates disparate streams of information about basic bodily regulation to inform us of *global organismic state* relative to predicted state. As Seth and Tsakiris (2018) point out, we feel the results of dehydration, poisoning or deoxygenation, but the effects are felt globally at the level of conscious awareness in sensations such as fatigue. When we attend to states such as thirst or fatigue, we do not succeed in more precisely representing the causal structure of the entities responsible for the experience (for example, the molecular mechanisms of dehydration or shifts in the production of metabolites and effects on neurotransmission). Rather, the goal is to establish the degree of departure from optimality of a global feeling state so that we can manage it at the systemic level (e.g., by drinking or resting). Interoceptive experience thus provides a personal-level proxy for the regulation of low-level homeostatic/allostatic variables whose mechanisms are opaque to introspection Joffily and Coricelli, 2013). The predictive or forecasting aspect is introduced by the need to regulate, by anticipating interoceptive fluctuations, and to evaluate actual state against those predictions. In other words, when we feel fatigue, we feel overall energy depletion relative to a prediction of optimal energy levels for that context. Interoceptive regulation uses experience to predict

“how an action would affect physiological homeostasis, given a model” (Seth and Tsakiris, 2018).

The idea that interoception is experienced as a systemic non-localized phenomenon connects with an interesting metaphysical point made by Wiese (2018) in his discussion of self-representation and predictive coding. Like Letheby and Gerrans (2017) and Hohwy and Michael, he argues that, phenomenologically, the self *seems* to be a substance: an enduring entity that underlies changing sensations and perceptions. Wiese points out, however, that interoceptive experience does not specify a particular localizable entity in a way that allows further discovery by the attentive deployment of perception or theoretical inference, because there is no concrete (by which he means spatiotemporally located) object of interoception to focus on. Wiese has an intricate and sophisticated predictive processing account of the sense of being the subject of experience as an “abstract enduring object” to which experience is *salient*. *Abstract* because it is a higher-order amodal integrator of lower-order information streams, and *enduring* because it represents the continuing entity in which those streams cohere.

“the apparent substantiality of the phenomenal self is explained by a structural feature of this salience model: it binds different dimensions of salience by representations of higher-order dimensions of salience (just as more abstract object representations bind representations of perceptual features in predictive processing accounts of feature binding)” (Wiese, 2018).

The similarity between material me, *qua* object of interoception, and objects of perception is their explanatory role as underlying hidden cause of coherence in experience. The difference between material me and objects of visual perception is the non-concreteness of material me. Seth and Tsakiris make a similar point to Weise.

“instrumental (control-oriented) interoceptive inference plausibly underlies a phenomenology related to the evaluation of the allostatic consequences of regulatory actions. *A non-localized, non–object-based phenomenology associated with both mood and emotion, and with the pre-reflective (i.e., non-reflexive) self-related experience of being an embodied organism* (Seth and Tsakiris, 2018).

## Affective Me: Emotion as Interoceptive Active Inference

So far, we have only explained why interoception creates “the pre-reflective self-related experience of being an embodied organism” as Seth and Tsakiris put it. The short answer is that allostatic regulation requires us to experience ourselves in interoception as an integrated entity to serve as the target of regulation. However, we have not begun to explain (i) how it is that interoception is associated with mood and emotion and (ii) how it is that this account can be mobilized to explain how people can feel detached from their bodily experiences in cases of depersonalization and pain asymbolia.

To do so, we need to explain the higher levels and dimensions of interoceptive self-modeling. The starting point is to note that some signals of body state are “vital signs.” Fatigue, sustained high temperature, or intractable nociception threatens the organism and requires urgent action. Consequently, we have evolved the capacity to feel such states, not simply as perturbations of body state, but as urgently motivating. Affective processes provide this “feeling of what matters” to slightly modify Antonio Damasio’s phrase.

Damasio’s account is a neo (William) Jamesian account of emotion, affect, and self-awareness that grounds all these experiences in bodily processing. The history of this idea and the way it is expressed are not uniform across the disciplines. Not everyone is using terms the same way. So, somewhat stipulatively, let me say I am using the term *self-awareness* to refer to a *feeling* of being the entity whose continued life underpins other forms of experience. I use the term *representation* to refer to information-bearing structures. There is no implication that the content of such structures is always consciously experienced. Thus, self-representation can be conscious or unconscious, but self-awareness is experienced. And I use emotion in a standard way derived from analysis of prototypical episodes of mental life (for example sadness, nostalgia, anger) that are evaluative and motivational, have characteristic bodily and behavioral indices, and are, typically, felt (Deonna and Teroni, 2012). I use the term *affect* to refer to a form of experience that carries emotional content. Thus, affect is common to emotion, which usually has an identifiable eliciting object, and mood, which does not. The feeling of anxiety can be part of an episode of emotion (anxiety about a specific forthcoming event) or, in the case of anxious mood, a feeling of hypervigilance and uncertainty without a particular object.

The idea that emotional episodes have evaluative, behavioral, cognitive, and affective components is part of the appraisal theory of emotion (Scherer, 2004; Grandjean et al., 2008). On this theory, emotional processes evaluate (appraise) the relevance of events (including internal events such as allostatic prediction error) for the organism. We might say that cognition and perception represent aspects of the world, and emotional processes represent the significance of that information for the well-being of the organism. And to do so, they need to model the organism as an entity with goals realized or frustrated in action (including internal regulatory action) (Scherer, 2004; Kalisch et al., 2006; Grandjean et al., 2008; Kalisch, 2009; Brosch and Sander, 2013).

This explains the subtle relationship between affective processing and interoception. Interoception integrates and aggregates allostatic variables to inform us of global organismic state. Emotional processes evaluate interoceptive signals against expectations about goal satisfaction in context. The result is experienced as an affective state. And affective states inherit from the interoceptive processes they metarepresent two interesting properties. They are intimately felt as states of a self, and at same time, they are global and non-localized. We do not experience sadness as a change in the state of a perceived object but as a global, overall feeling state of affective me. And in the same way as interoceptive experience is a proxy for allostatic regulation affective experience is a higher-level proxy for lower-level regulation. Experience or anticipation of danger, for example, prompts a suite of cognitive and behavioral responses that entrain a set of lower-level activities designed to optimize organismic function.

In order to provide the affective interpretation of changes in body state, affective processes exploit extra layers of emotional processing that metarepresent and interpret interoceptive signals (Stephan et al., 2016). The emotional interpretation of interoceptive signals requires integrating the interoceptive signal with information about the emotional salience of the situation and the subject’s affective history. In other words, “should material me expect to feel like this given the (emotional) context?” For predictive processing theories of emotion, predictive models of the emotional context set parameters that determine how physiological changes are regulated and experienced. Thus, interdependent models representing the emotional world (hostile, favorable, tractable *for me*) and the capacities of the organism to deal with that world interpret and predict interoceptive changes in a continuous cycle. It is in that sense that emotional processes are forms of interoceptive active inference (Barrett and Simmons, 2015; Barrett et al., 2016; Barrett, 2017; Kleckner et al., 2017). They provide higher-level interpretive and regulatory models for the reduction of interoceptive prediction error signals. Thus, emotional processes are part of a hierarchy of active inference. Interoception is allostatic active inference, and emotion is interoceptive active inference.

Still higher levels of self-modeling, narrative or conceptual, interpret and predict states of affective me, and one can see that other forms of self-representation and related experience, agential, and sensorimotor are guided and reinforced by their effects on affective me. In the end, without affective me, we are an organism to whom the world and its own states, as represented by our battery of cognitive faculties, no longer matter.

## Neural Correlates of Material and Affective Me: Emotional Transcription

Affective experience is produced by emotional processes that integrate interoceptive information with perception and cognition to produce the “feeling of what happens,” in Antonio Damasio’s phrase. To create this feeling, emotional processes effectively transcribe bodily feelings into affective experiences. This is why an interoceptive state such as fatigue can be experienced as disconsolate apathy when transcribed emotionally as part of an episode of sadness. As a bodily state, it has a particular experiential signature. Transcribed by emotional processes as the state of a self, rendered hopeless by an irretrievable loss, it has an affective signature. Given the way emotional and interoceptive processes are woven together, almost every interoceptive state is transcribed like this. It takes disorders and dissociations, such as pain asymbolia or rare states of emotional neutrality, to decompose their interactions. There is an analogy with delusions of misidentification (DMS) based on loss of predicted affective response to familiar faces. Normally, the feeling of familiarity evoked by recognizing a face is not salient amid the flux of experience, but when it is absent, the experience is of seeing a familiar but feeling “as if” one sees a stranger. Similarly, when one’s own body is damaged, the mind predicts an affective response. When that feeling is absent, it feels “as if” the experience is not happening to the subject. It is actually a striking parallel between disorders of depersonalization and the experiential (or first stage as it is sometimes called) component of DMS that they are reported in “as if” vocabulary (Breen et al., 2001; Brighetti et al., 2007; Coltheart et al., 2010). In both cases, the loss of predicted affective response combined with preserved cognition in other relevant domains (face recognition or nociception/interoception) creates the experience of estrangement.

The fatigue of depression and its morphing into disconsolate apathy provides a nice case study of the role of hierarchical interoceptive inference in producing affective me. Fatigue is an adaptive state designed to restore depleted subsystemic function. If, however, fatigue is intractably sustained, the result will be a persistent homeostatic/allostatic error signal experienced as a characteristic interoceptive state: weariness and exhaustion. The active inference hierarchy will exploit higher-level models to interpret and contextualize these interoceptive signals (Barrett et al., 2016; Friston et al., 2018; Velasco and Loev, 2020). Those higher-level models include models of affective me and narrative me that predict the effects on feeling state of activity across system, given the subject’s life history. Initially, such models predict restoration consequent on rest. If, however, the homeostatic error signals “from below” cannot be canceled, higher-level models can be revised to reflect that signal. This reflects the general principle that the hierarchical processing system settles into a state that minimizes error across the system. The self will be modeled as unable to control basic states and to act efficaciously in the world. At the level of affective me, the state is now felt as apathy and anhedonia, possibly anxiety at the prospect of exertion. At the level of narrative self-representation, thoughts of hopelessness and inadequacy can come to dominate. As Stephan et al., put it in their predictive processing account of fatigue and depression:

“belief of failure at one’s most fundamental task—homeostatic/allostatic regulation—… arises from experiencing enhanced interoceptive surprise. We suggest that fatigue is a (possibly adaptive) initial allostatic response to a state of interoceptive surprise; if dyshomeostasis continues, the belief of low allostatic self- efficacy and lack of control may pervade all domains of cognition and manifests as a generalized sense of helplessness, with depression as a consequence” (Stephan et al., 2016).

This quotation suggests a bottom-up etiology (“belief arises”), but active inference accounts such as this also give a constitutive role to top-down models in reconfiguring low-level allostatic processing. As Seth puts it:

“On this theory of interoceptive inference […], emotional states (i.e., subjective feeling states) arise from top-down predictive inference of the causes of interoceptive sensory signals […]” (Seth and Friston, 2016, p. 9).

The hierarchical nature of interoceptive processing and emotional processing is reflected in cytoarchitecture (Barrett and Simmons, 2015). Sections of the posterior insula cortex (PIC) take primary interoceptive afferents and integrate those representations to coordinate basic regulatory functions. The PIC, for example, integrates values of allostatic variables such as blood pressure and hydration as well as nociception (bodily damage). These signals are progressively remapped and integrated with other information at higher levels of cognition. Although it is not the only channel for bodily signaling, its role as a primary integrative hub of interoceptive afferents makes PIC a crucial substrate of the experience of material me (Singer et al., 2009; Medford and Critchley, 2010; Gasquoine, 2014; Moayedi, 2014).

The AIC is specialized to re-represent and integrate information about body state to allow us to feel the significance of interoceptive states as affects. AIC sits at the apex of the so-called “salience system,” the neural hierarchy that signals whether and how information matters to the organism. In order to perform its role, it must communicate with emotional processing hubs that coordinate appraisal of that information at all levels (Craig, 2009a; Craig, 2009b; Garfinkel and Critchley, 2013). The AIC is an enigmatic and functionally ubiquitous system widely connected to both lower- and higher-level processing circuitry. One telling feature is its consistent involvement in the processing of self-relevant information and the switching/anticorrelation between executive and default processing (a key substrate of narrative me) (Starr et al., 2009). Another is its connectivity to hubs of lower- and higher-level emotional appraisal (amygdala and ventromedial prefrontal cortex). These hubs coordinate the appraisal of and response to information presented in sensorimotor and higher-level cognition, respectively (Bechara et al., 1999; Scherer, 2004; Koenigs and Grafman, 2009; Adolphs, 2010; Pessoa and Adolphs, 2010; Gerrans and Scherer, 2013; LeDoux and Pine, 2016). Thus, the AIC is activated by perception of emotionally salient/self-relevant scenarios. Its activity allows us to feel the emotional significance of events. It is also active in scenarios in which we reflect on past events or anticipate the future, allowing so-called mental time travel to be imbued with affective significance. Thus, AIC activity can also provide the affective texture for anticipation and recollection as well as sensory processing. When imagining going on holiday or getting married, the affective texture of the episode predicts how the action will make us feel. This is a crucial adaptation for learning and planning. We plan our actions on behalf of future states of affective me. To do so, we remember how previous actions made us feel. This way of conceptualizing mental time travel makes it a form of extended active inference in which affective regulation is a proxy for overall organismic regulation (Suddendorf and Corballis, 2007; Boyer, 2008; Buckner et al., 2008; Broyd et al., 2009; Spreng et al., 2009; Carhart-Harris and Friston, 2010).

Thus, it is not surprising to see that contemporary affective neuroscience treats experience produced by AIC activation as a form of higher-order bodily representation that represents the integrated functioning of the organism *evaluated against emotionally salient goals creating a sense of self in the process* As Bud Craig (2009a) puts it:

“The integration successively includes homeostatic, environmental, hedonic, motivational, social, and cognitive activity to produce a ‘global emotional moment,’ *which represents the sentient self at one moment of time”* (Craig, 2009a).

In other words, affective me is the body under an emotional mode of presentation (to import some philosophical jargon). I am endorsing Craig’s idea that the AIC plays a key role in transforming interoceptive signals integrated by the PIC into representations of states of an affective self. This process can be described as one of higher-order metarepresentation and interpretation but to do so underplays constitutive influence of higher-order affective processing on lower-order processing. The role of emotional processing is not just to determine the relevance of interoceptive predictive error *after the fact* but also to set the parameters that determine which allostatic variations become prediction errors. How the bodily signal is processed depends on how it is emotionally contextualized.

Thus, I prefer to describe the relationship between AIC and PIC as emotional transcription. The AIC is an integrative hub of processes that transform a bodily into an affective signal, in the process transforming what would otherwise be a pure bodily feeling into an affective/emotional one. The AIC communicates with hubs of emotional processes to convert the neural signal representing interoceptive information into a neural signal representing the emotional significance of that information for the organism.

This transcription creates the crucial affective dimension of self-modeling. Without it, we could navigate the world using other dimensions, agential, narrative, sensorimotor, and bodily in order to optimize organismic functioning, however, we would lack a way to experience the *significance* of our interactions with the world.

Furthermore, this affective dimension of self-modeling provides a simple and effective proxy for the adaptive integration of other dimensions and the systems they coordinate. When our narrative, agential sensorimotor, agential, and bodily self-models are simultaneously optimized, we feel good: our organism is prospering in the world. When it is not, we feel a form of negative affect appropriate to the context. Within the predictive coding framework, negative affect signals failure to reduce prediction error across the system. More accurately, it reflects failure to reduce prediction at a rate predicted for that action (Joffily and Coricelli, 2013).

This is not a claim that the AIC is a discrete or modularized substrate of self-representation. Rather, its integrative role, connecting low- and high-level emotional processing and interoception and in the generation of affective experience, makes it an important hub of processing that enables us to feel the significance of events as affective states. Predictive coding suggests that the mind will model fluctuations in affective states by attributing them to a continuing entity: the thing that experiences the emotional ups and downs. Affective me is that entity. And a good candidate for its neural substrate, given its role as a hub of self-referential processing, is the AIC.

## Pain Asymbolia as a Form of Depersonalization Experience

The idea that the AIC is the substrate of affective self-modeling fits with studies of (relatively) selective damage or hypoactivity of AIC in disorders of depersonalization. In depersonalization disorder, patients report phenomenology such as the following:

“I feel some degree of ‘out of it’ all the time (…) I can sit looking at my foot or my hand and not feel like they are mine. This can happen when I am writing, my hand is just writing, but I’m not telling it to. It almost feels like I have died, but no one has thought to tell me. So, I’m left living in a shell that I don’t recognize any more” (Sierra and David, 2011).

Within the multidimensional multilevel framework, this could be explained as a result of preserved narrative/conceptual levels of self-modeling and basic bodily self-modeling in the absence of the basic experience of *being* the self who experiences autobiographical episodes. Another classic description of generalized depersonalization is Dugas’ patient who said:

“I only feel anger from the outside, *by its physiological reactions*” (Dugas and Moutier, 1911) my italics,” quoted in Billon (2017b).

This is a particularly telling example because it suggests that bodily processes and bodily awareness are intact, but the patient feels detached from them, despite awareness that they occur in his own body. On the account developed above, that is the result of failure to transcribe bodily interoceptive signals into affective.

The mechanism is sometimes hypothesized to be spontaneous inhibition of AIC by the ventrolateral prefrontal cortex (Medford et al., 2006; Medford, 2012; Medford et al., 2016). On most accounts, this is an involuntary defensive/dissociative response to unmanageable adversity. Of course, given the integrative role of the AIC and its dense multidirectional coupling and functional connectivity, there may be no unique cause of hypoactivity. What matters to the account here is that in the experience of depersonalization AIC hypoactivity is unpredicted by a self-model that anticipates AIC activity in context. It could be the case that the AIC is not receiving interoceptive afferents from lower levels or systems that appraise those signals for emotional relevance. If, however, the AIC is not responding in a predictable way to those afferents, the result is an error signal experienced as loss of predicted affect. The result is that most dimensions of self-representation, bodily, agential sensorimotor, and narrative are intact, but the agent does not feel as if any of the resultant experiences belong to her. The reason is that the AIC is no longer functioning to allow her to feel the significance of bodily changes evoked by her passage through the world (Gerrans, 2015, 2019). It is worth mentioning here that this interpretation of the role of the AIC has been disputed on the bases of cases of AIC lesion with “preserved emotional and affective responses” (Philippi et al., 2012; Damasio et al., 2013; Feinstein et al., 2016). My reading of these cases, however, is that the patients have intact *behavioral* aspects of emotion (such as aversive response) and primary interoception, which accounts for intact bodily feeling. However, the same patients do not seem to exhibit affective aspects of emotion such as feelings of sadness or remorse. Similarly, their empathic responses are cognitive rather than affective. In fact, the profile of Roger, the subject of discussion in two key articles, somewhat resembles that of Dugas’ patient (Gerrans, 2019). Very interestingly, Roger cannot be aversively conditioned to painful stimuli, although he responds aversively on each separate presentation is intact. This suggests that he does not anticipate negative affective experience when re-presented with the aversive stimulus.

Rather than discuss full-blown global depersonalization, this section concentrates on a fascinating subtype of depersonalization experience in which only one channel of processing is disconnected from affective me. Pain asymbolia is in which the subject feels pain or its nociceptive aspects, but says that the pain feels as if it does not matter or does not belong to her.

Colin Klein has argued persuasively that pain asymbolia is a form of depersonalization for pain. As he puts it:

“the phenomenology of asymbolia might resemble a kind of depersonalization syndrome. … The asymbolic, and the depersonalized more generally, feel sensations that they are estranged from—*that they do not take to be theirs in the sense that we normally do*. … [This]does show that there is another sense in which our sensations may be unified: as sensations over which we have a feeling of ownership. Asymbolia, and depersonalization more generally, shows *that this sort of unity may fail*. Its failure comes *not from a change in the sensations we feel, but in the sort of agents we are* (Klein, 2015) [my italics].

Klein suggests that the pain sensation is unchanged, but what has changed in the experience is “the sort of agents we are,” i.e., the type of self we are. This can be finessed still further when we add that this latter change must itself be experienced. Otherwise, the patient would not report the feeling that the pain sensation does not matter to *her.* Given the previous discussion, it is not quite right to say that the sensations have not changed, but the experience of the self has. Given that the self-model sets the parameters for bodily representation and consequent experience, a change in self-model affects the quality of experience.

Pain asymbolia is a nice example of the connection between basic bodily processing, emotional processing, and affect. Pain itself is a representation of damaged body state (nociception), but given its significance for organisms, the nociceptive signal is almost automatically appraised at primary level as distressing. Thus, pain, aversive response, and negative affect are very tightly linked (Krahé et al., 2013; Klein, 2015; Gogolla, 2017; Von Mohr and Fotopoulou, 2018; Gehrlach et al., 2019). Another way to put this is to say that bodily damage is represented at multiple levels in terms of its effects not just on the body but on the self and its prospects.

“Pain can therefore constitute a process of perceptual inference about nociceptive signals on the basis of predictive, top-down signals about the homeostatic significance of such signals in the context of other synchronous biological, cognitive, and social conditions. Furthermore, such re-mappings of interoceptive signals across the neurocognitive hierarchy suggest possible neurobiological mechanisms by which not only cognitive, but also social contextual factors can influence the awareness of interoceptive and other multimodal information about one’s own body” (Krahé et al., 2013).

Given these facts, the substrate of pain experience is a complex network of nociceptive, interoceptive, and social/cognitive/emotional circuitry. This “pain matrix” incorporates the insula as well as somatosensory and limbic regions (Starr et al., 2009). Characterizing the role of the insula in the matrix is complex due to its extensive connectivity, but one study reports a consensus view:

“insula may be well positioned to utilize cognitive information to modulate connected brain areas involved in processing of sensory-discriminative, affective, and cognitive-evaluative components of pain” (Wiech and Tracey, 2013).

or, as I might put it, to help coordinate higher-level active inference in response to lower-level nociceptive prediction error. And one feature of this coordinating role is the production of affective states that inform the organism of the significance of bodily damage. When, however (due to hypoactivity in the AIC), the predicted negative affect does not occur, the subject has to explain away the resultant prediction error. The bodily self-model is already functioning optimally: telling the organism that she is damaged. The narrative model is also intact: it says explicitly that pain should produce negative affect. Thus, the patient is in the situation of sensing bodily damage and knowing, intellectually, that she has bodily damage but feeling no distress in a situation in which she normally feels it automatically. She reports the result as the feeling that the pain is not “hers.” Pain asymbolics no longer assign or feel emotional significance in response to bodily damage in virtue of hypoactivity in their AIC. Effectively, they are in the situation of losing a crucial dimension of affective self-modeling for nociception. Consequently, they report that the experience is painful but that it does not matter and feels as if it is not happening to *them*. What this shows is that “mineness” can be lost locally, for aspects of bodily functioning, such as pain (Phillips et al., 2001; Phillips and Sierra, 2003; Medford et al., 2006; Simeon and Abugel, 2006; Simeon et al., 2008; Stein and Simeon, 2009; Sierra et al., 2012; Michal et al., 2013; Sedeño et al., 2014; Medford et al., 2016; Gogolla, 2017; Gerrans, 2019).

The idea that the anterior insula is a substrate of the feeling of mineness for pain via its role in affective processing is consistent with the similarity between depersonalization experience for pain and mild opioid analgesia. In opioid analgesia, patients report that the pain is not extinguished but *no longer matters*. A key finding here is that that opioids target not only the PIC, as one might expect, but also the AIC and related limbic structures involved in emotional processing.

The AIC in fact is even more responsive than PIC to low doses of opioids. This is an adaptation. It is easier for an organism to regulate emotional/affective response to bodily damage than to repair bodily damage. Thus, in contexts where the organism cannot devote resources to repair, it inhibits the system that produces negative affect and thereby stops pain from drawing attention away from other relevant activities. Opioids exploit this adaptation, down-regulating the AIC, reducing, not pain itself, but the felt significance of pain.

“the FMRI data suggest that opioid analgesics can directly influence emotional responses at low doses that do not alter sensory aspects of pain” (Lee et al., 2014).

Another way to put this is to say that mild opioid analgesia produces a mild form of pain asymbolia.

This suggestion about the role played by modulation of AIC activity independent of nociception is supported by an interesting finding about *voluntary imagination of sensory states*.

“For the visualization of internal state sensations, this meant increased activity in areas of interoceptive sensory processing, including the mid and anterior insula in the right hemisphere. This is a critical finding, as it suggests that primary interoceptive cortex, located in the posterior insula, was not significantly involved in the imagery of internal state sensations” (Bennett and Baird, 2009).

Cases like this suggest that when we imagine or reflect on an experience, we (re)construct the affective component of experience. In other words, we represent not what happens to us but how it matters to us. We empathize with our past or future self by activating circuitry, which represents not body state *per se*, but the significance of body state. This allows us to enrich the narrative self-model with episodic and affective imagery, transforming it from a linguistic autobiographical model to one which we feel, as well as know, is ours. In other words, activity in the AIC links the affective dimension of self-modeling to our bodily and narrative models. In this respect, the affective self-model has a crucial integrative role. It sits at the border between sensorimotor bodily modeling, which controls organismic interaction with the environment, and explicit top-down cognition, which exploits narrative and conceptual models. It allows us to feel not just like a cognitive system manipulating a body through the world (which we are) but a self, with an autobiographical trajectory that matters.

## Conclusion

The mind models and predicts fluctuations of affect by attributing them to a continuing self. That “self-model” allows us to experience not just the way things are, but the way they matter to us given our history, goals, and concerns.

Perhaps the most crucial dimension of self-modeling is affective. The ability to feel the significance of our engagements with the world allows us to regulate our organism moment to moment and offline over long time scales to “feel the future” and rehearse the past. In this sense, affective processes knit systemic functioning together allowing us to pursue organismic well-being by regulating our affective states.

When this integrative process fails (due to hypoactivity in the AIC or systems that link affective processing to cognition), but the world and body are being otherwise accurately represented, the subject feels that something is wrong. Furthermore, her autobiographical knowledge is undisturbed. The result is a massive prediction error in the hierarchical multidimensional self-model. The narrative and bodily dimensions are intact, but the predicted affective dimension is absent. She reports the result naturally enough by saying that the experience feels as if it is not happening to her.

## Data Availability Statement

All datasets generated for this study are included in the article/supplementary material, further inquiries can be directed to the corresponding author/s.

## Author Contributions

The author confirms being the sole contributor of this work and has approved it for publication.

## Conflict of Interest

The authors declare that the research was conducted in the absence of any commercial or financial relationships that could be construed as a potential conflict of interest.
